# Symmetric Objects Become Special in Perception Because of Generic Computations in Neurons

**DOI:** 10.1177/0956797617729808

**Published:** 2017-12-08

**Authors:** R. T. Pramod, S. P. Arun

**Affiliations:** Centre for Neuroscience and Department of Electrical Communication Engineering, Indian Institute of Science, Bangalore, India

**Keywords:** visual perception, visual search, object recognition, inferior temporal, symmetry

## Abstract

Symmetry is a salient visual property: It is easy to detect and influences perceptual phenomena from segmentation to recognition. Yet researchers know little about its neural basis. Using recordings from single neurons in monkey IT cortex, we asked whether symmetry—being an emergent property—induces nonlinear interactions between object parts. Remarkably, we found no such deviation: Whole-object responses were always the sum of responses to the object’s parts, regardless of symmetry. The only defining characteristic of symmetric objects was that they were more distinctive compared with asymmetric objects. This was a consequence of neurons preferring the same part across locations within an object. Just as mixing diverse paints produces a homogeneous overall color, adding heterogeneous parts within an asymmetric object renders it indistinct. In contrast, adding identical parts within a symmetric object renders it distinct. This distinctiveness systematically predicted human symmetry judgments, and it explains many previous observations about symmetry perception. Thus, symmetry becomes special in perception despite being driven by generic computations at the level of single neurons.

Symmetry is a salient visual property. People appreciate it in nature and create it in art. It influences a variety of fundamental perceptual phenomena (Bertamini & Makin, 2014; Treder, 2010; Wagemans, 1995, 1997). In behavior, symmetry is easy to detect (Baylis & Driver, 1994, 2001; Corballis & Roldan, 1975; Friedenberg & Bertamini, 2000; Makin, Rampone, Pecchinenda, & Bertamini, 2013; Wagemans, 1995) and remember (Kayaert & Wagemans, 2009). Symmetry influences figure-ground organization (Baylis & Driver, 1994; Devinck & Spillmann, 2013) and improves 3-D reconstruction (Vetter, Poggio, & Bülthoff, 1994). In the brain, high-level visual areas show stronger responses to symmetric than to asymmetric objects (Makin et al., 2013; Palumbo, Bertamini, & Makin, 2015; Sasaki, Vanduffel, Knutsen, Tyler, & Tootell, 2005; Tyler et al., 2005), and perturbing them affects symmetry judgments (Bona, Cattaneo, & Silvanto, 2015; Bona, Herbert, Toneatto, Silvanto, & Cattaneo, 2014; Cattaneo, Mattavelli, Papagno, Herbert, & Silvanto, 2011). These studies demonstrate that symmetry has a special status, yet they do not elucidate why this is so.

To address this issue, we drew on the finding that the neural response to a whole object can be reliably predicted as the sum of responses to each of its parts (Sripati & Olson, 2010a; Zoccolan, Cox, & DiCarlo, 2005). We hypothesized that when identical parts are present in an object (making it symmetric), they will interact nonlinearly and cause the response to that object to deviate systematically from the sum of the responses to its parts. We created a set of objects in which two arbitrarily chosen parts were connected by a stem (Fig. 1a) and targeted single neurons in the monkey inferior temporal (IT) cortex, an area critical for recognition. To relate these neural representations to behavior, we performed parallel psychophysical experiments in humans using the same stimuli. Our main finding is a remarkable null result: Responses to symmetric objects showed no systematic deviation from part summation and were no different from responses to asymmetric objects according to any other response measure in neurons. Yet symmetric objects were more distinctive from each other, which we demonstrate is due to part summation itself. This neural distinctiveness predicts symmetry perception in humans and also explains a variety of observations in the literature.

**Fig. 1.**
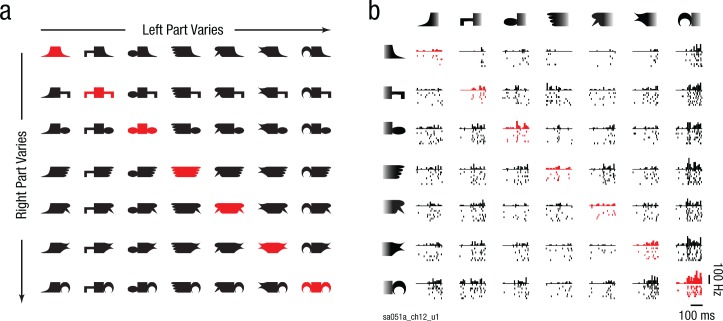
Stimuli (a) and example responses in monkey inferior temporal (IT) cortex (b). Each neuron was tested with horizontally and vertically oriented versions of these shapes, but results are shown here for horizontal objects for ease of exposition (for vertical objects, see Section 1 in the Supplemental Material). Each object was created by attaching two parts on either side of a stem. Objects along a row or column share the same part on the right or left side, respectively. Symmetric objects are highlighted here in red only for the purposes of illustration; asymmetric objects are shown in black. All objects were presented in white against a black background. The plot in (b) shows responses of an example IT neuron (with neuron ID at the bottom left) to the stimuli in (a). Stimuli are sorted in order of increasing response along rows and columns. Each plot consists of a histogram depicting the number of spikes in successive 20-ms bins and raster plots depicting spike times elicited during individual trials across the entire 200-ms image-presentation period.

## Method

### Monkey neurophysiology

All animal experiments were performed according to a protocol approved by the Institutional Animal Ethics Committee of the Indian Institute of Science and the Committee for the Purpose of Control and Supervision of Experiments of Animals, Government of India. Surgical procedures and other experimental details were identical to those described in previous reports from our laboratory (Ratan Murty & Arun, 2017).

#### Behavior

Monkeys were trained to perform a fixation task. Each trial began with the appearance of a fixation dot (diameter = 0.2°), and after successful fixation, a series of seven images appeared on the screen. Each image lasted for 200 ms and was followed by a 200-ms interstimulus interval. On successfully maintaining gaze inside a 3° window around the fixation dot throughout the trial, the animal received a juice reward. Although the fixation window was relatively large, post hoc analysis of eye position during correct trials revealed that the gaze was closely centered on fixation (*SD* = 0.27° and 0.35° along horizontal and vertical axes, respectively).

#### Single-unit recordings

We recorded neuronal activity in the left anterior IT cortex of two adult male monkeys (denoted Ka and Sa) using a 24-channel multicontact electrode (U-Probe, Plexon, Dallas, TX). For details of recording sites, refer to our previous study (Ratan Murty & Arun, 2017). Continuous waveforms were analyzed off-line and sorted into clusters using spike-sorting software (OfflineSorter, Plexon). This yielded 180 visually responsive neurons that were used for all subsequent analyses (93 from Ka and 87 from Sa). All the key results described were qualitatively similar in both monkeys.

#### Stimuli

We chose seven parts and created 49 two-part objects by placing the parts on either side of a stem (Fig. 1a). This design resulted in 42 asymmetric and 7 symmetric objects. Each neuron was tested using the same shapes in a horizontal and vertical orientation, with the latter obtained by rotating horizontal stimuli counterclockwise by 90°. All stimuli measured 4° along the longer dimension with each part subtending 1.33°. In all, there were 98 stimuli tested for each neuron (49 horizontal and 49 vertical objects). We obtained qualitatively similar results for both horizontal and vertical objects. For ease of exposition, results for horizontal objects are described here in the main text, and results for vertical objects are detailed in the Supplemental Material available online.

#### Trial design

Each trial consisted of one symmetric and six asymmetric objects presented one at a time, with the constraint that no two objects in a trial shared a part at the same location on the object (this was done to avoid response adaptation). Horizontal and vertical objects were presented in blocks of seven trials each (i.e., after one repetition of all 49 objects). Trials in which the animal broke fixation were repeated after a random number of other trials. In this manner, we collected neural responses to at least eight repetitions of each stimulus.

### Human behavior

All human subjects had normal or corrected-to-normal vision, were naive to the purpose of the experiments, and gave consent to an experimental protocol approved by the Institutional Human Ethics Committee of the Indian Institute of Science, Bangalore, India.

#### Experiment 1 (visual search)

A total of 8 subjects (age = 20–30 years; 5 female, 3 male) participated in this experiment. This number was chosen because this sample size yielded visual search times with extremely high split-half consistency in our previous studies (Pramod & Arun, 2014, 2016). The stimuli consisted of the 49 horizontally oriented objects used in the neuronal recordings. Subjects were seated approximately 60 cm from a computer monitor controlled by custom MATLAB programs (The MathWorks, Natick, MA) written using the Psychophysics Toolbox (Brainard, 1997). Each trial began with a fixation cross at the center of the screen, displayed for 500 ms. Following this, a 4 × 4 search array containing one oddball item among multiple identical distractors was presented; a red vertical line ran down the middle of the screen to facilitate left/right judgments. Each item measured 3° along the longer dimension (slightly smaller than the 4° size used in the neural recordings). Items were centered at the grid locations but jittered in position in both *x* and *y* directions by ±0.45° according to a uniform distribution to prevent alignment cues from guiding search. Subjects were instructed to indicate the side on which the oddball target appeared as quickly and accurately as possible by pressing a key (“Z” for left and “M” for right). They had to make a response within 10 s of the onset of the search array or the trial was aborted. Response time for trials with correct responses was used for subsequent analyses. Trials on which errors were made were repeated randomly later in the task. The data from this experiment have been reported previously (Pramod & Arun, 2016), but the analyses reported here are unique to this study.

#### Experiment 2 (symmetry task)

A total of 18 subjects (age: 19–41 years; 3 female, 15 male) participated in this experiment (none of them had participated in Experiment 1). We chose this sample size because it yielded response times with good split-half consistency in our previous studies (Mohan & Arun, 2012). The stimuli were the same 49 horizontally oriented objects used in the neural recordings. On each trial of this task, a fixation cross was shown for 750 ms at the center of the screen, followed by a horizontal object stimulus with the longer dimension measuring 4° (similar to the dimensions used in the neural experiment). The stimulus was briefly flashed on the screen for 200 ms, after which a noise mask measuring 4° × 4° was presented for 4,800 ms or until the subject made a response. Subjects were asked to report whether the briefly presented stimulus was symmetric or asymmetric using the “S” or “N” key, respectively. The response time (measured from the onset of the stimulus) for each trial was used for analyses. To eliminate response bias resulting from unequal numbers of symmetric and asymmetric objects, we presented each symmetric object 24 times and each asymmetric object 4 times. Thus, subjects performed 168 trials (7 objects × 24 repeats) with symmetric objects and an equal number of trials with asymmetric objects (42 objects × 4 repeats), resulting in a total of 336 correct trials. Error trials (either a wrong response or failure to respond within 5 s of stimulus onset) were repeated after a random number of other trials. To avoid any effects of familiarity, we used only the data from the first four trials with both symmetric and asymmetric objects for all analyses.

#### Experiment 3 (symmetry and visual search with 64 objects)

This experiment consisted of two tasks—visual search and categorization. Eight subjects (age: 22–33 years; 1 female, 7 male) participated in both tasks, and an additional 4 subjects (age: 21–31 years; 3 female, 1 male) participated only in the categorization task. We chose these sample sizes because in previous studies from our lab on visual search (Pramod & Arun, 2014, 2016) and object categorization (Mohan & Arun, 2012), they yielded data with high split-half consistency. We created a set of 32 symmetric and 32 asymmetric objects, with the constraint that every symmetric object shared one part with one of the asymmetric objects. This ensured that subjects could not use the memorized identity of any single part to determine that an object was symmetric. We chose 32 parts of varied complexity: Some objects contained two discernible parts (like those in Experiment 1), and others were simpler shapes, such as circles and squares, with no discernible parts. Each subject performed a visual search task followed by a symmetry task. In the visual search task, the trials consisted of symmetric objects as targets and asymmetric objects as distractors or vice versa. In all, there were 1,024 pairs of objects (32 symmetric objects × 32 asymmetric objects). Each subject performed two correct search trials involving each pair with either item as the target. Thus, we collected visual search data for 2,048 trials (1,024 pairs × 2 repetitions) from each subject. All other details were similar to those in Experiment 1, except that the objects measured 4° along the longer dimension. All details of the symmetry task were identical to those in Experiment 2, except that each object was presented 4 times, bringing the total number of correct trials to 256 (64 objects × 4 trials per object).

### Data analysis

#### Part-sum model

We avoided testing neural responses to isolated parts because isolated parts can contain extra features that may qualitatively alter the response. Instead, our stimulus set consisted of all possible combinations of seven parts on either side, which allowed us to estimate the underlying response to each part assuming linear summation. Since all seven parts could appear independently in either location on the object, we estimated the contribution of each part on the left or right side independently. In all, we modeled the whole object response as a linear sum of 15 possible regressors (7 parts × 2 locations and a constant term). The resulting set of 49 equations can be summarized as the matrix equation ***y* = *Xb***, where ***y*** is a vector of 49 whole-object normalized responses (all responses were divided by the maximum response), ***b*** is a vector of 15 unknown part activations, and ***X*** is a 49 × 15 matrix whose rows contain 0s and 1s indicating whether a particular part is present (1) or absent (0) in the corresponding objects. To fit the model, we used standard linear regression (using the *anovan* function in MATLAB).

#### Pixel and V1 models

In the pixel model, each pixel was considered a feature, and the corresponding gray level was the feature value. Each 51-by-152-pixel image was converted to a 7,752-dimensional-feature vector of pixel gray-level values. In the V1 model (Pinto, Cox, & DiCarlo, 2008; Ratan Murty & Arun, 2015), each image was represented as a vector of outputs of a bank of Gabor filters tuned to eight orientations and six spatial frequencies. In addition, each Gabor filter had input contrast and output divisive normalization. In all, the V1 model resulted in a 372,096-dimensional-feature representation for each image. For both pixel and V1 representations, distance between images was calculated as the Euclidean distance between the feature vectors.

#### Prediction of behavioral dissimilarity using neural dissimilarity

To estimate how behavioral dissimilarities measured using visual search in humans matched with neural dissimilarities measured in monkey IT neurons, we tried two approaches. First, we directly compared the neural dissimilarity between every pair of objects (calculated as the average firing-rate difference elicited by the two objects) with the behavioral dissimilarity in visual search (calculated as the reciprocal of the average search time for the two objects). This revealed a moderate correlation (*r* = .34, *p* < .00005) that could be potentially biased if some parts elicited little or no activity across neurons. To resolve this issue, we fitted a model in which the behavioral dissimilarity for each pair of objects was a weighted sum of the neural dissimilarities across neurons. This amounted to solving a linear regression of the form ***y*** = ***Xb***, where ***y*** is a vector of behavioral dissimilarities for all object pairs, ***X*** is a matrix containing absolute differences in firing rate elicited by each pair across neurons, and ***b*** is an unknown vector specifying the contribution of each neuron to behavior. This model yielded excellent fits to the neural data.

#### Consistency of symmetry detection times

To estimate an upper bound on the ability of models to predict symmetry detection times, we calculated the split-half correlation between the average response times of two randomly chosen groups of subjects. However, this number underestimates the true reliability of the data since it is based on two halves of the data. To estimate the true reliability of the data, we corrected the split-half correlation using a Spearman-Brown correction, given as *r_c_* = 2*r*/(1 + *r*) where *r_c_* is the corrected correlation, and *r* is the split-half correlation. This corrected correlation is reported throughout as *r_c_*.

#### Calculation of distinctiveness using visual search

We calculated distinctiveness of an object as its average dissimilarity from other objects. To predict symmetry detection times in Experiment 2, we used the visual search data from Experiment 1. For each object, we calculated its distinctiveness as its average dissimilarity from other 48 objects tested in the experiment.

In Experiment 3, measuring search dissimilarities was experimentally impossible since the total number of possible objects using these parts was too large (32 × 32 = 1,024 objects), and the number of pairwise dissimilarities was even larger (^1024^C_2_ = 523,776 pairs). Accordingly, we collected search data for only 1,024 object pairs containing one symmetric and one asymmetric object and used a computational model described previously (Pramod & Arun, 2016) to estimate all possible dissimilarities. According to this model, the total dissimilarity between two objects AB and CD is given by a sum of part-part comparisons at corresponding, opposite, and within-object locations. Since all the part-part relations were strongly correlated, we fitted a part-sum model using only corresponding-part terms to estimate the underlying part relations. This amounts to solving a matrix equation ***y*** = ***Xb***, where ***y*** is a vector of 1,024 observed search dissimilarities, ***X*** is a 1,024 × 496 matrix of 1s and 0s indicating the presence (1) or absence (0) of each possible pair of parts, and ***b*** is a vector of 496 (^32^C_2_) part-part dissimilarities. Having estimated all pairwise part dissimilarities at corresponding locations, we used the scaling relations observed previously to predict the full set of dissimilarities as *y* = *X****b*** + *aX_a_****b*** – *wX_w_****b***, where *X_a_* and *X_w_* are the corresponding matrices for the opposite-location and within-object comparisons, and *a* and *w* are scalar values that represent the relative contributions of the corresponding terms. This modified model yielded comparable predictions of the observed data (*r* = .94, *p* < .000005) compared with the model containing only corresponding parts (*r* = .94, *p* < .000005). However, it captures additional features present in the data, such as mirror confusion and distinctiveness of symmetric objects, both of which require across-object and within-object part relations. To calculate the distinctiveness of each object, we determined the average dissimilarity between this object and all 1,023 other objects using the model-predicted dissimilarities.

#### Simulation of artificial population of neurons

From the neural data, we predicted that symmetric objects would be more dissimilar than asymmetric objects purely because of part summation. To test this prediction, we created a population of 50 artificial neurons and calculated the neural responses to 7 symmetric and 42 asymmetric hypothetical objects. Specifically, each artificial neuron in the population had randomly initialized part selectivity (uniformly distributed from 0 to 1) that was identical on both sides of the object. The neural response to all 49 objects was then computed simply as the sum of part responses. Neural dissimilarities for each pair of objects was calculated as the average absolute difference in firing rates elicited by the two objects.

## Results

We recorded from 180 neurons in the IT cortex of 2 monkeys while they viewed horizontally oriented symmetric and asymmetric objects made by joining two arbitrary shapes (Fig. 1a). The responses of an example IT neuron are shown in Figure 1b. This neuron had similar preferences for parts at both locations and responded strongest to objects with its preferred part at either end. Importantly, its responses to symmetric and asymmetric objects did not differ (average firing rate from 0 to 200 ms after stimulus onset: 16.0 and 15.3 Hz for symmetric and asymmetric objects, respectively; *p* = .65, rank-sum test across 7 symmetric and 42 asymmetric objects). This was true across all neurons as well: Symmetric objects did not elicit greater responses (average firing rate: 15.6 and 15.5 Hz for symmetric and asymmetric objects, respectively; *p* = .49, sign-rank test on average firing rates across neurons).

### Can the neural response to the whole object be explained as a sum of responses to its parts?

**Fig. 2.**
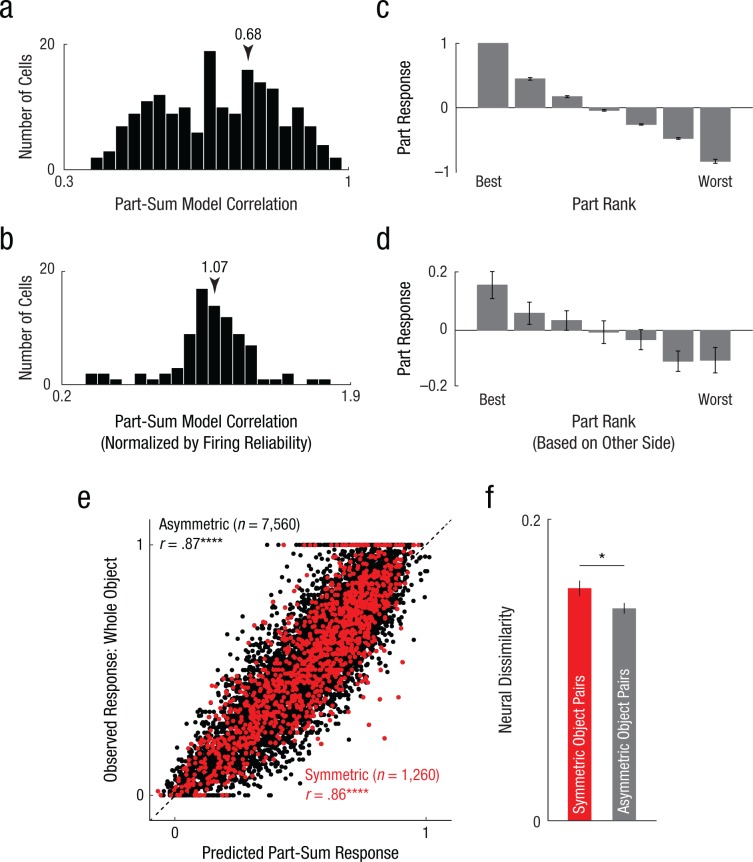
Part summation in symmetric and asymmetric objects in monkey inferior temporal (IT) neurons. The histogram in (a) shows results across neurons for the correlation between the observed response and the response predicted by the part-sum model. The response of each neuron was modeled as a sum of part activations on each side. To estimate the degree to which the model captures the systematic variation in firing for each neuron (b), we fitted the part-sum model to odd-numbered trials, calculated its predictions on even-numbered trials, and divided the resulting model correlation by the observed correlation between odd and even trials. The plot depicts the normalized model correlation calculated in this manner for all neurons with significant split-half correlations (*n* = 87). The arrows in (a) and (b) indicate the average correlation coefficient. The graph in (c) shows part response (normalized to the maximum) for left or right parts for neurons showing at least one main or interaction effect (*n* = 128). The graph in (d) shows normalized part response for the right parts arranged according to the left-part preference (and vice versa). Error bars in (c) and (d) show ±1 *SEM*. The scatterplot (e) shows the normalized observed response plotted against the response predicted by part summation across all objects and neurons for asymmetric objects and symmetric objects. The dashed line represents the least-square fit. Asterisks indicate significant correlations (****p < .00005). The bar graph in (f) shows the mean neural dissimilarity for symmetric object pairs and equivalent asymmetric object pairs. The asterisk indicates a significant difference between pair types (*p < .05). Error bars represent ±1 *SEM* calculated on neurons (*n* = 180).

Because we created a large number of whole objects using a small number of parts, we were able to ask whether the neural response of each neuron could be modeled as a sum of part activations (see the Method). We avoided recording responses to isolated parts because isolated parts contain extra features (where they are separated from the whole object) that make them qualitatively different than when they are embedded within an object (Pramod & Arun, 2016). The resulting part-sum model yielded excellent fits to the neural response for the example neuron shown in Figure 1b (*r* = .93, *p* < .000005). It also yielded a significant correlation for all neurons (Fig. 2a; average *r* = .68 across 180 neurons).

This correlation between the observed and predicted response could be low either because the part-sum model did not explain all the systematic variation in firing across neurons, or because neural firing itself was noisy. To assess these possibilities, we calculated the correlation between the predictions of the model trained on odd-numbered trials and the observed firing rate on even-numbered trials. We then compared this with the degree with which the firing rate estimated from odd-numbered trials predicted the firing rate on even-numbered trials. A perfect model would show a correlation roughly equal to the reliability of firing. The ratio of model correlation and split-half firing correlation, which we defined as *normalized correlation*, then represented the degree to which the part-sum model explains the systematic change in firing. The normalized correlation was on average close to 1 across reliable neurons (Fig. 2b; normalized correlation: *M* = 1.07, *SD* = 0.25 for 85 neurons). We note that some normalized correlations were larger than 1, implying that model fits were better than the split-half reliability itself. On closer investigation, we found that these occurred in neurons with noisy firing (average split-half reliability: .59 for 35 neurons with normalized correlation < 1, .44 for 52 neurons with normalized correlation > 1, *p* = .03, rank-sum test), suggesting that the spuriously high normalized correlation arose from poor firing reliability. We conclude that the part-sum model accurately explained nearly all the systematic variation in firing rate across neurons.

Next, we asked whether neurons showed consistent selectivity for part shape at both locations. To assess this possibility, we first identified neurons that showed significant part modulation by performing an analysis of variance (ANOVA) on the firing rate of each neuron across trials with left part (seven levels) and right part (seven levels) as factors. We then selected a subset of 128 neurons that showed at least one significant main or interaction effect. Next, we ranked the estimated part response (from the part-sum model) on a given side (left or right) from best to worst and plotted the average normalized response across neurons (Fig. 2c). This plot depicts the selectivity of the neuron to any given side. To assess whether this selectivity was similar on the other side, we calculated the normalized response for each neuron for parts on the other side (right or left) ranked in the same order as before. If neurons showed inconsistent selectivity for parts on both sides of the object, the response to parts on one side would not change systematically when ranked according to the part preference on the other side. Instead, part responses decreased systematically when ranked according to part preferences on the other side (Fig. 2d). This average slope was significantly different from zero (average slope = −0.043, *p* < .00005 on a sign-rank test across 128 neurons). Thus, a part that elicits a strong response on one side also elicits a strong response on the other side. We conclude that IT neurons showed similar part selectivity at both locations in the object.

### Do symmetric objects deviate more than asymmetric objects from part summation?

Next, we asked whether the match between observed and predicted responses was different between symmetric and asymmetric objects. Contrary to our expectations, the match between the observed and predicted responses was no worse for symmetric objects (Fig. 2e; model vs. data correlation: *r* = .86 and .87 for symmetric and asymmetric objects, respectively, *p* < .00005 in both cases). Likewise, the residual error was no different for symmetric objects (average absolute error between observed and predicted responses: .093 and .096 for symmetric and asymmetric objects, respectively, *p* = .64, rank-sum test across the average absolute error across 180 neurons).

The above analyses were based on fitting the part-sum model to all objects and may have been subject to overfitting. To rule out this possibility, we fitted the part-sum model to a subset of the data and tested it on the data that were left out. Specifically, we selected 35 asymmetric objects to train the model and tested it on the remaining 14 left-out objects (7 symmetric and 7 asymmetric objects). For each neuron, we repeated this analysis 6 times so that each asymmetric object was used only once in the left-out set. Even here, the residual error for symmetric and asymmetric objects in the left-out set was not different across neurons (average residual error: .14 for symmetric objects and .14 for asymmetric objects, *p* = .33, sign-rank test on the average residual error across 180 neurons). We conclude that both symmetric and asymmetric objects are equally subject to part summation.

### Are symmetric objects more distinctive from each other?

The above analyses show that symmetric and asymmetric objects are both subject to part summation with very little nonlinear interactions. In other words, symmetric objects have no special status in terms of how their parts combine. We then wondered whether symmetric objects have any special status in terms of how they relate to each other or to other objects.

To examine this possibility, we calculated the neural dissimilarity between pairs of objects using the average difference in normalized firing rate across neurons and compared the neural dissimilarity between symmetric objects (which differ in two parts; *n* = 21) and between matched asymmetric objects that also differ in two parts (*n* = 420). Note that including all asymmetric object pairs would result in an artificially low dissimilarity because of including pairs of objects with shared parts, which have low dissimilarity. Comparing symmetric and asymmetric object pairs differing in two parts, we found a significant difference across the recorded neurons (average normalized neural dissimilarity: .159 and .145 for symmetric and asymmetric object pairs, *p* = .011, sign-rank test across average symmetric pair vs. asymmetric pair distances across neurons; Fig. 2f).

This difference was also present in both animals considered separately (average normalized dissimilarity: .17 and .15 for symmetric and asymmetric pairs, respectively, *p* = .04, sign-rank test across 93 neurons in Ka; .15 and .14 for symmetric and asymmetric pairs, respectively, *p* = .03, sign-rank test across 87 neurons in Sa). It was also present when we calculated the raw neural dissimilarity (average raw firing-rate difference: 4.7 and 4.3 spikes per second for symmetric and asymmetric object pairs; *p* = .012, rank-sum test). While these firing-rate differences appear relatively small, it is not uncommon for relatively large effects in behavior to manifest as a relatively small difference in firing rates across the population (Baker, Behrmann, & Olson, 2002; Kayaert, Biederman, & Vogels, 2003; McMahon & Olson, 2007; Sripati & Olson, 2010b). In sum, we conclude that symmetric objects are more distinctive from each other compared with asymmetric objects at the neural level.

### Are symmetric objects more distinctive even in behavior?

**Fig. 3.**
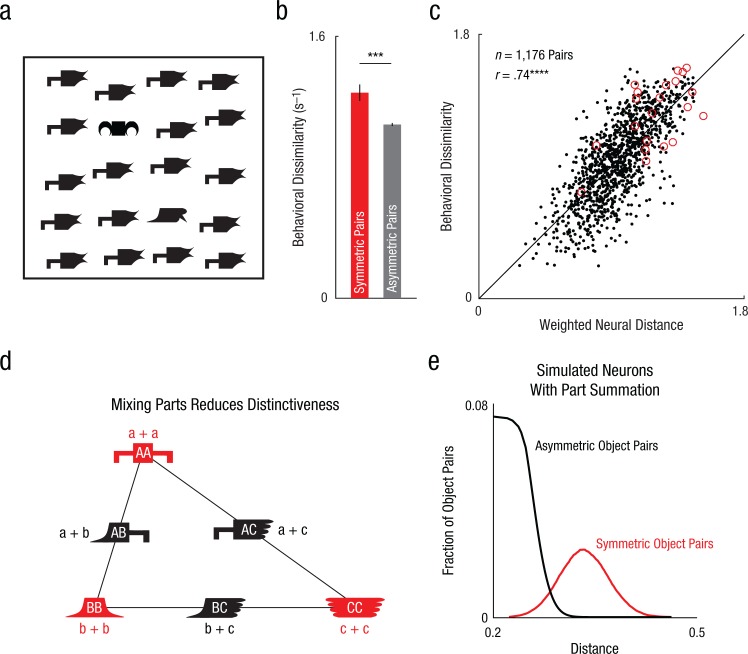
Distinctiveness of symmetric objects as a result of part summation. The example search array from Experiment 1 (a) contains a symmetric oddball and an asymmetric oddball embedded among asymmetric distractors. The symmetric object is easier to find than the asymmetric object, even though both objects differ in two parts from the distractors. The bar graph (b) shows mean behavioral dissimilarity for symmetric object pairs and equivalent asymmetric object pairs measured using visual search in humans (Experiment 1). The asterisks indicate a significant difference between pair types (***p < .0005). Error bars represent ±1 *SEM* calculated on object pairs (*n* = 21 for symmetric objects; *n* = 420 for asymmetric objects). The scatterplot (c; with best-fitting regression line) shows behavioral dissimilarity for each pair of objects (in humans; Experiment 1) plotted against weighted neural dissimilarity across inferior temporal (IT) neurons (in monkeys), separately for symmetric object pairs (circles) and asymmetric object pairs (dots). The asterisks indicate a significant correlation (****p < .00005). The schematic (d) illustrates how part summation results in greater distinctiveness. Let parts A, B, and C evoke neural activity represented by vectors a, b, and c. According to part summation, the response to any object AB will be a + b. As a result, the symmetric objects AA, BB, and CC will evoke activity 2a, 2b, and 2c, whereas the asymmetric objects AB, BC, and AC will evoke activity a + b, b + c, and a + c. The response to each asymmetric object pair (e.g., AB) will lie at the midpoint of the line joining the two symmetric object pairs (e.g., AA and BB). As a result, the asymmetric objects AB, BC, and AC will evoke more similar activity than symmetric objects AA, BB, and CC. In other words, combining different parts in an object reduces its distinctiveness, just like mixing paints, whereas combining similar parts in an object maintains the original distinctions between the parts. This simple property causes symmetric objects to be farther apart in general than asymmetric objects, while producing no net difference in the average response to symmetric and asymmetric objects. To confirm that this extends to many neurons with heterogeneous selectivity, we created 50 artificial neurons with identical part responses at both locations, but with random part selectivity, and used them to generate whole-object responses. The plot (e) shows the distribution of distances for symmetric object pairs and asymmetric object pairs.

The above results show that symmetric objects tend to be dissimilar compared with asymmetric objects at the level of monkey IT neurons. To establish the behavioral correlate of this effect in humans, we performed a visual search experiment (Experiment 1) using the same shapes. On each trial, subjects saw a search array containing one oddball among identical distractors (as in Fig. 3a) and searched for all possible pairs of stimuli across trials. We calculated the reciprocal of search time as a measure of behavioral dissimilarity to compare with neural dissimilarity (Arun, 2012). We then compared the behavioral dissimilarity for symmetric and asymmetric object pairs as before and found a similar result: Symmetric objects were more distinctive from each other compared with asymmetric objects with unique parts (average dissimilarity for horizontal objects: 1.26 for symmetric objects vs. 1.07 for asymmetric objects, *p* = .00017, rank-sum test across 21 symmetric object pair and 420 asymmetric object-pair distances; Fig. 3b).

Having shown similar results in behavior and neurons, we asked whether the behavioral dissimilarity measured in humans could be explained by the neural dissimilarity observed in IT neurons across all stimuli. Behavioral dissimilarity was moderately correlated with neural dissimilarity (*r* = .34, *p* < .00005). This correlation could be biased because of neural sampling, for example, if very few neurons responded to some parts because of variations in their shape selectivity. To address this issue, we fitted a simple linear model in which behavioral dissimilarity between each pair of stimuli was given by the weighted sum of firing-rate differences across neurons (see the Method). This weighted neuronal model yielded a strong correlation between behavioral and neural dissimilarity (*r* = .74, *p* < .00005; Fig. 3c). We conclude that symmetric objects are more distinct from each other than asymmetric objects, both in single neurons and in human visual search.

### Are symmetric objects more distinctive in low-level image representations?

The above results show that symmetric objects are more distinctive from each other compared with equivalent asymmetric objects, but this could arise directly from the image pixels or from low-level visual processing. To rule out these possibilities, we compared symmetric and asymmetric object representations in two computational models: a pixel-based model analogous to the retina and a V1 model matched to the properties of primary visual cortex (Pinto et al., 2008; Ratan Murty & Arun, 2015). We concatenated the output of each model and calculated pairwise distances between symmetric and asymmetric stimuli as before. These distances were not significantly different in both models (average distances in the pixel-based model for symmetric and asymmetric pairs: 0.86 and 0.86, *p* > .6; average distances in the V1 model for symmetric and asymmetric pairs = 27.5 and 27.5; *p* > .6, rank-sum test across 21 symmetric object pairs vs. 420 asymmetric object pairs). Thus, the greater distinctiveness of symmetric objects is an emergent property of high-level representations and is not a trivial consequence of the input image or of early visual processing.

### Why are symmetric objects more distinctive?

We have shown that symmetric objects have no special status at the neural level in terms of how their parts combine, yet they attain a special status by becoming distinctive from each other. How does this occur? Consider, for instance, a population of neurons activated by parts A, B, and C with response vectors **a**, **b**, and **c** containing the responses evoked by each neuron (Fig. 3d). Then, by part summation, the responses to the symmetric objects AA, BB, and CC will be the vectors 2**a**, 2**b**, and 2**c**, whereas the responses to asymmetric objects AB, BC, and AC will be the vectors **a** + **b**, **b** + **c**, and **a** + **c**. Because the vector **a** + **b** lies exactly at the midpoint of the vectors 2**a** and 2**b**, it follows that these vectors will always have a specific arrangement within a plane, as depicted in Figure 3d. It can be easily seen that the asymmetric objects AB, BC, and AC will be closer together, whereas AA, BB, and CC remain as far apart as their constituent parts were originally. In other words, just as when mixing paints, adding identical parts maintains distinctiveness, whereas mixing diverse parts diminishes it. Note that the average response to symmetric and asymmetric objects remains the same (at the centroid of the triangle) even though their average dissimilarity is different. The only requirement for this to work is similar selectivity for parts at either location, a form of position invariance that is true for IT neurons (Fig. 2b; Sripati & Olson, 2010b). To confirm this prediction further, we simulated an artificial population of neurons with identical part selectivity at both locations and created responses to symmetric and asymmetric objects. Distances between symmetric and asymmetric objects belonged to clearly different distributions (Fig. 3e).

### Does distinctiveness explain symmetry perception in humans?

**Fig. 4.**
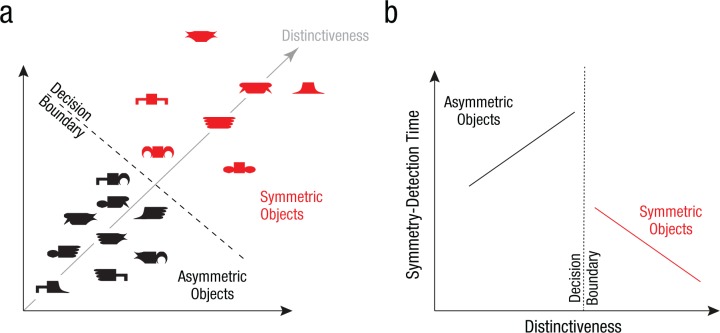
Hypothetical relation between distinctiveness and symmetry perception. Part summation causes symmetric objects to become more distinctive in the underlying neural representation (a) compared with asymmetric objects. If this property drives symmetry perception, symmetry judgments should be determined at least in part by a decision boundary that separates symmetric and asymmetric objects on the basis of their distinctiveness. If distinctiveness is the underlying quantity driving symmetry detection, then objects close to the decision boundary should be the hardest to judge during symmetry detection (b), whereas objects far away from the boundary should be easy. Specifically, this hypothesis predicts that symmetry detection time should decrease as symmetric objects become more distinct but should increase as asymmetric objects become more distinct. Note that this is qualitatively different from the pattern expected from salience: Salient objects would produce faster responses for both symmetric and asymmetric objects.

Thus far, we have shown that symmetric and asymmetric objects are both governed by part summation, part summation results in symmetric objects becoming more distinct, and these effects are present both in monkey IT neurons as well as in human visual search. This is depicted schematically in Figure 4a. Could this neural property influence symmetry perception?

Specifically, if symmetry perception is based on neural distinctiveness, there must be a criterion level of distinctiveness at which objects that exceed this criterion are classified as symmetric, and those that fall below it would be classified as asymmetric. This, in turn, implies fast and accurate symmetry judgments for objects far away from this criterion and slow responses for objects close to this criterion, according to common models of decision making (Ashby & Maddox, 2011; Mohan & Arun, 2012). Put differently, symmetric objects that are more distinctive should elicit a fast response, whereas asymmetric objects that are more distinctive should elicit a slow response (Fig. 4b). We performed two behavioral experiments on human subjects to test this prediction. Note that this prediction is qualitatively different from the pattern expected from salience: Salient objects should elicit faster responses regardless of their symmetry (as we confirmed—see Section 4 in the Supplemental Material).

**Fig. 5.**
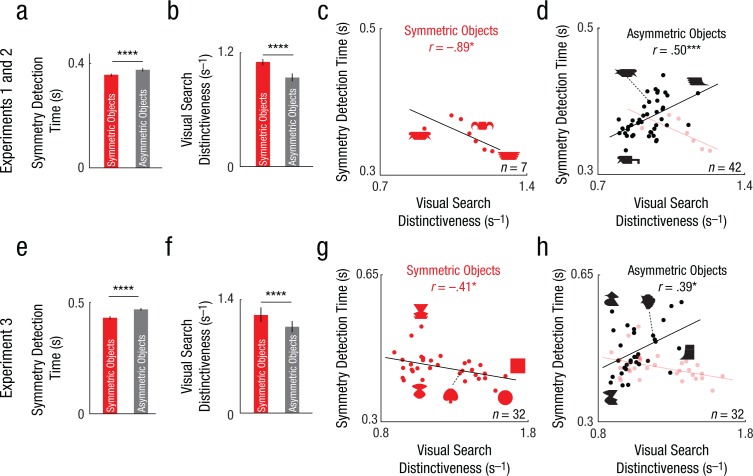
Distinctiveness as a predictor of symmetry perception in Experiments 1 through 3. Average response time during the symmetry-judgment task (a) is shown as a function of object type in Experiment 2. Average distinctiveness in visual search (b) is shown as a function of object type in Experiment 1. The scatterplots (with best-fitting regression lines) show the relation between symmetry detection time and visual search distinctiveness, separately for (c) symmetric objects and (d) asymmetric objects in Experiment 2. In (d), results for symmetric objects are overlaid faintly for comparison. Average response time during the symmetry-judgment task (e) and average distinctiveness in visual search (f) are shown as a function of object type in Experiment 3. The scatterplots (with best-fitting regression lines) show the relation between symmetry detection time and visual search distinctiveness, separately for (g) symmetric objects and (h) asymmetric objects in Experiment 3. In (h), results for symmetric objects are overlaid faintly. In the bar graphs, error bars represent ±1 *SEM* across objects, and asterisks indicate significant differences between object pairs (****p < .00005). In the scatterplots, asterisks indicate significant correlations (*p < .05, ***p < .0005).

In Experiment 2, subjects performed a standard symmetry-judgment task using the same 49 stimuli as in the previous experiments. Subjects were faster to judge an object as symmetric than to judge it as asymmetric (Fig. 5a; average response times: 354 ms and 377 ms for symmetric and asymmetric objects; *p* = .00002 for the main effect of symmetry in an ANOVA on response times with subject and symmetry as factors). We then used the visual search dissimilarity from the earlier experiment (Experiment 1) to calculate the distinctiveness of each object as its average dissimilarity relative to all other objects. Distinctiveness also was significantly different between symmetric and asymmetric objects (Fig. 5b; mean distinctiveness = 1.11 and 0.94 s^–1^ for symmetric & asymmetric objects; *p* < .005, rank-sum test on average distinctiveness for 7 symmetric and 42 asymmetric objects). Importantly, response times in the symmetry-judgment task were negatively correlated with distinctiveness for symmetric objects (Fig. 5c; *r* = −.89, *p* = .012) and positively correlated for asymmetric objects (Fig. 5d; *r* = .50, *p* < .0005). These correlations approached the consistency of the data itself (see the Method; *r_c_* = .91 and .53 for symmetric and asymmetric objects, *p* < .05).

The above findings are all based on testing a small group of objects. To confirm the generality of our findings, we performed an additional behavioral experiment (Experiment 3) using a much larger group of objects, which included unitary objects, such as circles and squares, with no discernible parts. As before, subjects performed a symmetry-judgment task on a set of 32 symmetric and 32 asymmetric objects (created using 32 possible parts) and a visual search task to measure perceptual dissimilarities. In the symmetry-detection task, subjects responded faster to symmetric objects than to asymmetric objects (Fig. 5e; average response time: 431 ms for symmetric objects, 470 ms for asymmetric objects; *p* < .000005 for the main effect of symmetry in an ANOVA on response times with subject and symmetry as factors). In the visual search task, subjects performed 1,024 searches involving all possible symmetric-asymmetric object pairs. However, calculating the distinctiveness posed a problem: In the previous experiment (Experiment 2), we were able to estimate the average distinctiveness of each object by averaging its dissimilarity with all 48 other objects in the set. However, this was simply not feasible here because it requires measuring 1,023 (32 × 32 − 1) dissimilarities for each object. Instead, we fitted the data to a part-sum model (see the Method), used the model to predict all ^1024^C_2_ pairwise dissimilarities, and used them to calculate the distinctiveness of each object relative to all other objects. Model-predicted distinctiveness was again significantly larger for symmetric objects (Fig. 5f; mean distinctiveness = 1.21 and 1.06 s^–1^ for symmetric and asymmetric objects; *p* < .00005, rank-sum test on 32 symmetric object distinctiveness scores vs. 32 asymmetric object distinctiveness scores). Importantly, as we predicted, response times in the symmetry-judgment task were negatively correlated with distinctiveness for symmetric objects (Fig. 5g; *r* = −.41, *p* = .02) and positively correlated for asymmetric objects (Fig. 5h; *r* = .39, *p* = .03). These correlations were close to the consistency of the data itself (*r_c_* = .48 and .59 for symmetric and asymmetric objects). These correlations were weaker but remained significant even when distinctiveness was calculated purely on the basis of taking the average dissimilarity of each object with the 63 other objects tested in the experiment (*r* = −.44, *p* = .013 for symmetric objects and *r* = .36, *p* = .045 for asymmetric objects). However, we note that there are some asymmetric and symmetric objects that elicited similar response times and also showed similar distinctiveness, indicating that distinctiveness by itself may not explain all of symmetry perception (see the Discussion).

To summarize, in human behavior, distinctiveness speeds up the response to symmetric objects but slows down the response to asymmetric objects. These patterns are consistent with the possibility that distinctiveness strongly influences symmetry perception.

## Discussion

In this study, we set out to investigate symmetry in visual objects using a combination of neural recordings from monkey IT neurons and matched behavioral experiments in humans. Contrary to our initial expectations, symmetric objects did not show deviations from part summation or nonlinear part interactions. Instead, symmetric objects became distinctive as a direct consequence of part summation in neurons. This distinctiveness accurately predicted human symmetry judgments. Thus, the special status of symmetry in perception is driven by generic computations at the neural level that make symmetric objects distinctive. Below, we discuss and reconcile the existing literature in relation to our findings.

### Symmetry and distinctiveness

Our finding that symmetry can be explained by a generic computation in neurons is consistent with the idea that symmetry perception is automatic and graded (Bertamini & Makin, 2014; Wagemans, 1997). However, there may be two distinct mechanisms that operate during symmetry detection, particularly in tasks such as detection of regular dot patterns (Wagemans, 1995). The first mechanism is automatic and graded, resulting in extremely fast responses to overall symmetry. We propose that this process is driven by distinctiveness. The second mechanism, which may involve local scrutiny and pattern matching across the image, may be initiated only when the overall pattern is not distinctive enough. The facts that distinctiveness explains most but not all of the variance in symmetry responses, and that some symmetric and asymmetric objects have the same distinctiveness, imply that both fast and slow processes are involved. However, our results place limits on the contribution of the second process. Understanding the second mechanism will require using patterns equated for distinctiveness so as to rule out the contribution of the first mechanism.

Our proposal that distinctiveness underlies symmetry perception offers a possible explanation for why symmetry judgments slow down with contour complexity (Kayaert & Wagemans, 2009): As a contour becomes more complex, its disparate parts undergo part summation, making them less distinctive. Conversely, simple contours contain many similar features that also undergo part summation, causing them to remain distinctive. This explanation requires that part summation occurs at multiple scales. Indeed, throughout the ventral visual pathway, the response to multiple stimuli in the receptive field is roughly equal to the average of the individual responses, a phenomenon known as divisive normalization (Carandini & Heeger, 2011; Sripati & Olson, 2010a; Zoccolan et al., 2005). This explanation also requires that neurons respond similarly to parts at multiple locations and across mirror reflection, properties that are certainly present in monkey IT (Connor, Brincat, & Pasupathy, 2007; Rollenhagen & Olson, 2000) and its homologue, human lateral occipital complex (Dilks, Julian, Kubilius, Spelke, & Kanwisher, 2011; Grill-Spector, Kourtzi, & Kanwisher, 2001). Thus, symmetric objects may become distinct only in high-level visual areas where receptive fields are large and invariant enough for part summation to benefit symmetry. This explains why only high-level visual areas show differential responses to symmetry (Sasaki et al., 2005; Tyler et al., 2005) and causally affect symmetry judgments (Bona et al., 2014, 2015; Cattaneo et al., 2011). It is also supported by our computational analysis showing that symmetry is not distinctive in the retinal image or models of low-level visual cortex.

The finding that symmetry perception can be partially explained using neural distinctiveness raises several potential problems. First, if distinctiveness drives symmetry perception, it would predict that any distinctive object can be potentially mistaken as being symmetric. This was indeed the case in our experiments, where some objects took unusually long times to be judged as asymmetric even though they did not appear anywhere close to being symmetric (Fig. 5). Second, could distinctiveness simply be bottom-up salience? While this is consistent with the fact that distinctive symmetric objects elicit faster responses, it is contradicted by the fact that distinctive asymmetric objects elicit slower (not faster) responses. In a separate experiment, we further confirmed that changing salience (by altering image contrast) produces uniformly slower responses for both symmetric and asymmetric objects (see Section 4 in the Supplemental Material). Thus distinctiveness is qualitatively different from salience. Third, it might be argued that a novel object may elicit a larger neural response (Meyer, Walker, Cho, & Olson, 2014; Woloszyn & Sheinberg, 2012), making it distinctive. However, it might not be mistakenly identified as being symmetric provided that its distinctiveness results from comparisons with other objects that share its parts or with other neurons representing the same object. This is consistent with norm-based accounts in which object responses are based on referencing an underlying average (Leopold, Bondar, & Giese, 2006; Leopold, O’Toole, Vetter, & Blanz, 2001).

### Relation to symmetry responses in the brain

Our results offer a novel interpretation of previous observations regarding brain responses to symmetry. Stronger neural responses to symmetric objects have been observed in blood-oxygen-level-dependent (BOLD) activations over extrastriate visual areas (Sasaki et al., 2005; Tyler et al., 2005). This could arise from symmetric objects being more distinctive from each other, leading to lower BOLD signal adaptation and consequently larger signal levels. Stronger responses to symmetry have also been observed in event-related potential studies (Makin et al., 2013; Palumbo et al., 2015). However, these differences arise relatively late (~400 ms after stimulus onset), consistent with symmetric objects attracting bottom-up attentional modulation because they are distinctive and consequently salient. Finally, stronger neural responses to symmetric than asymmetric objects have been observed in IT cortex of monkeys performing a symmetry-judgment task (McMahon & Olson, 2007). However, this facilitation may have arisen later in the response, which is consistent with attentional modulation arising because symmetric objects are distinctive or because they are task-relevant targets.

### Vertical versus horizontal symmetry

It is well known that symmetry about the vertical axis is detected fastest compared with other symmetries (Bertamini & Makin, 2014). Our argument for symmetric objects being more distinct is based on neurons showing similar part selectivity at both locations in an object. Thus, the faster detection of symmetry in horizontal objects may ultimately arise from more consistent part selectivity across locations within these objects.

To assess this possibility in our data, we first established that humans detect vertical symmetry faster than horizontal symmetry for our stimuli (see Section 2 in the Supplemental Material). While part responses are indeed more consistent for horizontal compared with vertical objects (cf. Fig. 2d and Fig. S2d), this difference was not robust. This discrepancy is difficult to interpret, especially given the absence of behavioral data in the monkey experiments and other experimental limitations (see Section 3 in the Supplemental Material). Evaluating these possibilities will therefore require further study. We propose that differences in symmetry detection across contour reflections and translations arise ultimately from intrinsic differences in generalization of shape tuning across these manipulations.

## Supplementary Material

Supplementary material

Supplementary material
