# Supplementary material for: Symmetric Objects Become Special in Perception Because of Generic Computations in Neurons
Source: Psychol Sci. 2017 Dec 8;29(1):95–109. doi: 10.1177/0956797617729808 (PMC5772447; doi:10.1177/0956797617729808)
Supplement: Supplementary material [file ArunSupplementalMaterial_rev.doc]

**SUPPLEMENTARY MATERIAL**

**for**

**Symmetric objects become special in perception due to generic computations in neurons**

**CONTENTS**

1. **NEURAL RESPONSES TO VERTICAL OBJECTS**
2. **VERTICAL VS HORIZONTAL SYMMETRY IN HUMANS**
3. **VERTICAL VS HORIZONTAL SYMMETRY IN IT NEURONS**
4. **EFFECT OF SALIENCE ON SYMMETRY PERCEPTION**

**SECTION 1. NEURAL RESPONSES TO VERTICAL OBJECTS**

The results in the main text were described for horizontally oriented objects but we obtained qualitatively similar results for vertically oriented objects, as detailed in this section.

**Can the response to the whole object be predicted as the sum of its parts?**

The responses of the Figure 1b neuron to the set of vertical objects is shown in Figure S1b. This neuron showed similar selectivity for top and bottom parts. Its response was no different for symmetric and asymmetric objects (average firing rate during 50-200 ms: 22.1 & 24.3 Hz for symmetric & asymmetric objects, p = 0.69, unpaired t-test across 7 symmetric and 42 asymmetric objects). This was true for the entire neural population for vertical objects (average firing rates: 15.92 & 15.81 Hz for symmetric & asymmetric objects, p = 0.21, sign-rank test on average firing rates across neurons).

We then asked whether neural responses to vertical objects can be explained using a sum of responses to parts. As before this model yielded excellent predictions of the observed whole-object responses for all cells (Figure S2a; average model correlation across 180 neurons: r = 0.66, all correlations p < 0.05). On calculating the normalized correlation as before, we found that the part sum model explained nearly all the systematic variation in firing rate (Figure S2b, average normalized correlation: 1.04 across 85 neurons with significant split-half correlation in firing). As before neurons with normalized correlation greater than 1 showed more noisy firing (average split-half correlations: 0.51 for 39 neurons with normalized correlation > 1, 0.59 for 45 neurons with normalized correlation < 1, p = 0.02, rank-sum test).

Next we asked whether neurons showed similar part selectivity at both locations. To assess this possibility we calculated the normalized part response from best to worst for one side (Figure S2c) and plotted the normalized part response for parts on the other side in the same order (Figure S2d). Part responses decreased systematically when ranked according to part preference on the other side, although this trend was not as clear as for horizontal objects (Figure 2d). The average slope of the part selectivity on the other side was significantly less than zero (average slope = -0.053, p < 0.000005 using a sign-rank test on slope of part selectivity curve across 123 neurons). We conclude that IT neurons show similar part selectivity for top and bottom parts.

**Do symmetric objects deviate more from part summation?**

As with horizontal objects, there was no difference in model performance for symmetric and asymmetric objects (Figure S2e; model vs data correlation: r = 0.88 & 0.87 for symmetric and asymmetric objects, both correlations significant at p < 0.00005; average absolute error: 0.096 & 0.099 for symmetric and asymmetric objects respectively, p = 0.49 on a rank-sum test across average absolute error for 180 neurons, with absolute error averaged across objects for each neuron).

**Relation to human visual search**

As before we found that behavioural dissimilarity measured on humans doing a visual search task was easily explained by a weighted sum of neural distances (Figure S3f; r = 0.71, p < 0.00005). As with horizontal objects, neural distances were significantly higher for pairs of symmetric objects than for pairs of asymmetric objects (Figure S3g; average normalized neural dissimilarity: 0.16 & 0.15 for symmetric and asymmetric object pairs, p = 0.03, sign-rank test across median symmetric pair vs asymmetric pair distances across neurons). The same pattern was true for behavioural dissimilarities (Figure S3h; average dissimilarity for vertical objects: 1.34 for symmetric objects vs 1.18 for asymmetric objects, p = 0.0007, unpaired t-test across 21 symmetric object pair & 420 asymmetric object pair distances).

We conclude that vertically oriented symmetric objects also conform to part summation just as well as asymmetric objects, yet become more distinctive due to part summation.

**
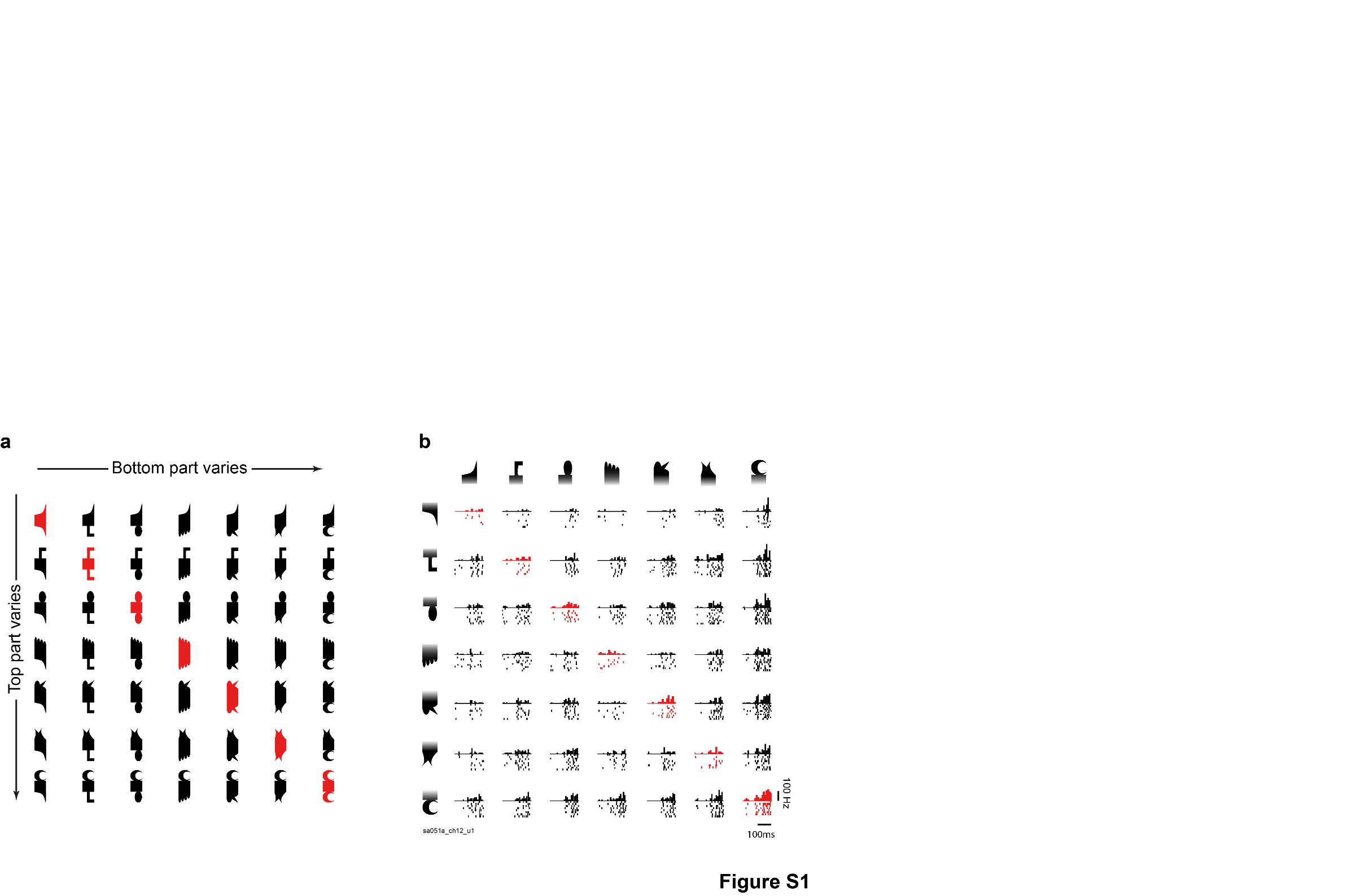
**

**Figure S1: Example neuronal response to vertical objects**

1. Set of vertically elongated objects used in the study. Objects along a row or a column share the same part on the top or bottom respectively. Symmetric objects are highlighted in red.
2. Responses of the neuron in Figure 1B to vertical objects.

**
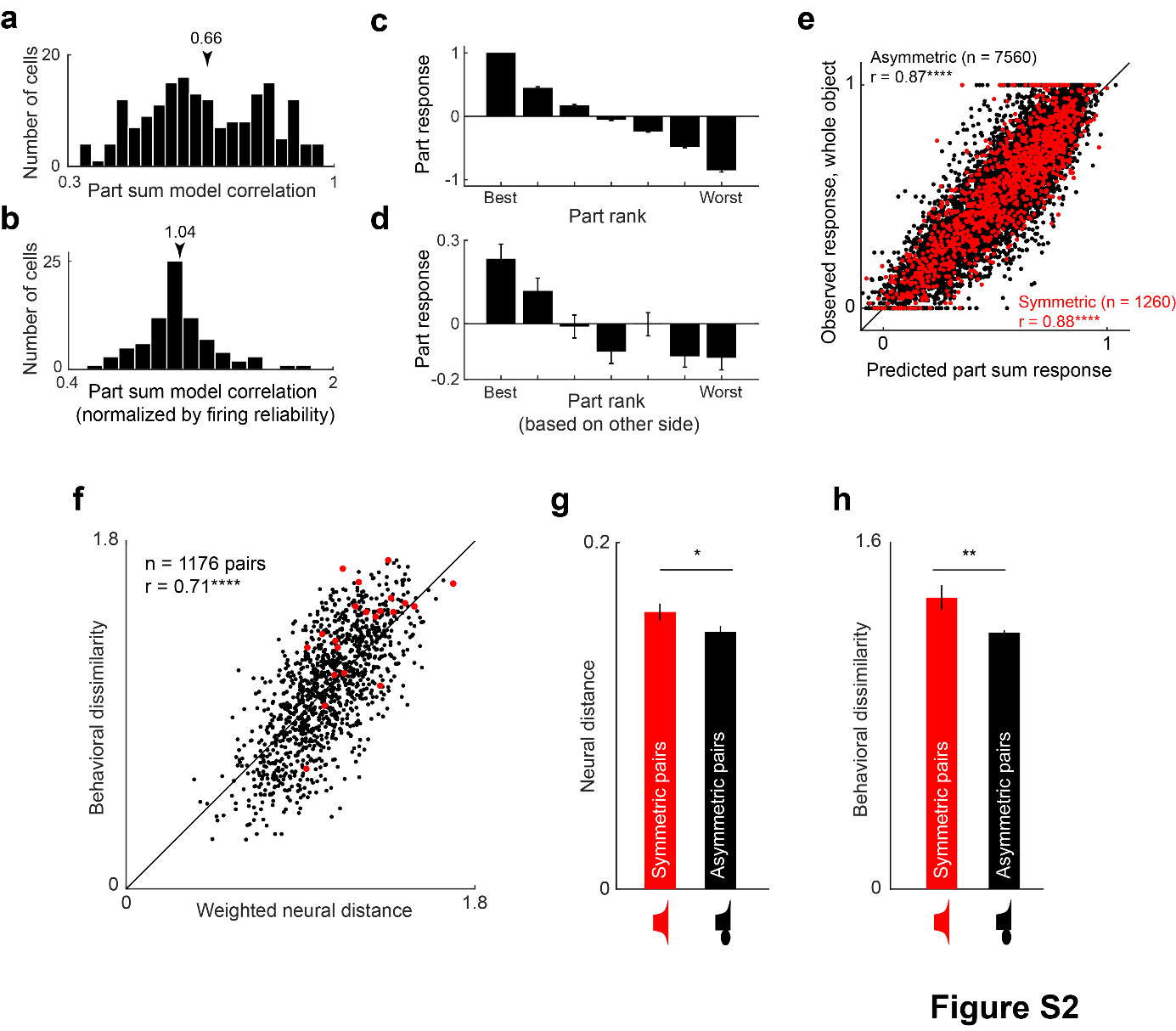
**

**Figure S2: Neural representation of symmetry in vertical objects**

1. Histogram across neurons of correlation between the observed response and the response predicted by the part sum model. The response of each neuron was modelled as a sum of part activations on each side. All 180 recorded neurons showed a significant model correlation, and the *arrow* represents the average correlation coefficient.
2. To estimate the degree to which the model captures the systematic variation in firing for each neuron, we fit the part sum model to odd-numbered trials and calculated its predictions on even-numbered trials, and divided the resulting model correlation by the observed correlation between odd and even trials. The plot depicts the normalized model correlation calculated in this manner for all neurons with significant split-half correlation (n = 85). The average normalized correlation is nearly 1, suggesting that the part sum model explains most of the systematic firing rate variation across trials. The fact that the normalized correlation exceeds 1 indicates that the split-half correlation is only a noisy estimate of the explainable variance in firing.
3. Part response (normalized to the maximum) for top or bottom parts ranked from best to worst for neurons showing at least one main or interaction effect (n = 123).
4. Normalized part response for the top parts arranged according to the bottom part preference (and vice-versa). The consistent decrease in response in this plot shows that parts that elicit strong response on one side also elicited a strong response on the other side.
5. Observed response plotted against the response predicted by part summation across all objects and neurons for asymmetric (*black dots*) and symmetric objects (*red dots*). Dotted line represents the least square fit line. Here and in subsequent figures, asterisks represent statistical significance: * is p < 0.05, ** is p < 0.005 etc. The dotted line represents the y = x line and the solid line represents the best-fitting line.
6. Behavioural dissimilarities measured using visual search in humans plotted against weighted neural distance from monkey IT neurons for vertical objects. Symmetric object pairs are shown in red.
7. Average neural distance between pairs of symmetric objects (*red*) and pairs of asymmetric objects (*black*). Error bars represent s.e.m calculated on neurons (n = 180).
8. Similar plot as in (g) but for behavioural dissimilarity. Error bars represent s.e.m calculated on object pairs (n = 21 for symmetric objects and n = 420 for asymmetric objects).

**SECTION 2. VERTICAL VS HORIZONTAL SYMMETRY IN HUMANS**

Previous studies of human symmetry perception have shown that symmetry about the vertical axis is easier to detect compared to symmetry about the horizontal axis. To establish this effect for our stimuli, we performed an additional behavioural experiment on humans in which we compared horizontal and vertical symmetry perception.

**Method**

A total of 8 subjects (aged 19-43 years, one female) participated in this experiment. The set of stimuli comprised of objects used in Experiments 2 & 3 of the main text. Specifically, we chose 7 vertical-axis symmetric and 7 asymmetric objects from experiment 2 (also used in the neuronal experiment); together with 32 vertical-axis symmetric and 32 asymmetric objects from experiment 3. We created the horizontal-axis symmetric and asymmetric objects by rotating the vertical-axis objects by 90 degrees in the counter-clockwise direction. Thus, we created a total of 156 objects (39 symmetric & 39 asymmetric objects each oriented either horizontally or vertically). All experimental procedures were identical to Experiments 2 & 3, with the only exception being the number of trials in the task. Subjects performed 4 correct trials for each object resulting in a total of 624 correct response trials. Symmetric/asymmetric and horizontal/vertical object trials were randomly interleaved.

**Results**

Subjects were moderately consistent in their responses in the task (split-half correlation of response times: *r* = 0.46, *p* = 0.0014 for horizontal objects and *r* = 0.43, *p* = 0.0003 for vertical objects). Subjects were faster to judge an object as symmetric than to judge it as asymmetric irrespective of object orientation (Figure S3; average response times for horizontal objects: 359 ms & 406 ms for symmetric and asymmetric objects; vertical objects: 377 ms & 418 ms for symmetric and asymmetric objects; p < 0.00005 for the main effect of symmetry in an ANOVA on response times separately for each object orientation with subject and symmetry as factors). Symmetry response times were significantly correlated between horizontal and vertical objects (r = 0.35, p = 0.027 for symmetric objects; and r = 0.62, p < 0.00005 for asymmetric objects).

We then asked whether subjects detected symmetry faster in horizontal objects (which are symmetric about the vertical axis). This was indeed the case: subjects were slower on vertical objects overall (average response times: 382 ms & 398 ms for horizontal and vertical objects; p = 0.003 for the main effect of orientation in an ANOVA on response times with subject, symmetry and object orientation as factors; p < 0.00005 for main effect of symmetry; p = 0.59 for interaction between symmetry and orientation). This difference was largely due to significant differences in response times for symmetric objects (average response times: 359 and 377 ms for horizontal and vertical symmetric objects; p < 0.005 for main effect of orientation in an ANOVA on symmetric object responses with subject and orientation as factors). In contrast, for asymmetric objects this difference was present but did not attain statistical significance (average response times: 406 & 418 ms for horizontal and vertical asymmetric objects; p = 0.13 for main effect of orientation in an ANOVA on response times with subject and orientation as factors).

We conclude that vertical-axis symmetry is easier to detect than horizontal-axis symmetry.

**Figure S3 – Horizontal vs vertical symmetry perception.** Average response times during symmetry judgment for horizontal and vertical objects. Red and black bars correspond to symmetric and asymmetric objects respectively. Error bars indicate s.e.m calculated across objects.

**SECTION 3. VERTICAL VS HORIZONTAL SYMMETRY IN IT NEURONS**

We have shown that symmetric objects become more distinct because of part summation in neurons. This argument rests critically upon neurons showing similar part selectivity at both locations in an object. This in turn predicts that that the faster detection of symmetry in horizontal objects by humans (observed in the preceding Section 2) is ultimately due to their neurons showing more consistent part selectivity at both locations for horizontal objects.

To assess this possibility, we compared the consistency of part selectivity across IT neurons for horizontal and vertical objects (Figure 2d vs Figure S2d). It can be seen that part responses decrease more systematically on average for horizontal objects (Figure 2d) compared to vertical objects (Figure S2d). Indeed, the correlation between part responses on both sides was stronger for horizontal compared to vertical objects (r = 0.97 for horizontal, r = 0.94 for vertical). To assess the significance of this difference we calculated the correlation between part responses on both sides for each neuron, and asked if the correlation was stronger for horizontal objects. This revealed no significant difference across neurons with significant ANOVA effects (average correlation across 104 neurons with at least one significant effect in both horizontal and vertical objects: 0.20 & 0.26 for horizontal and vertical, p = 0.35, sign-rank test). We also observed no significant difference in part correlation even across 30 neurons that showed both main effects of parts for horizontal as well as vertical objects (average correlation: 0.39 & 0.55 for horizontal and vertical). We conclude that part selectivity is equally consistent for both horizontal and vertical objects in IT neurons.

The lack of difference between horizontal and vertical objects in monkey IT neurons can be due to a variety of reasons. First, it could be that there is no advantage of horizontal symmetry in monkeys. This can only be established in monkeys performing a symmetry detection task. Second, given the contralateral bias in receptive fields, it is possible that horizontal objects were not positioned within the receptive field of each neuron as effectively as were vertical objects, which might have abolished any bias. Testing these possibilities will require precise measurements of receptive field and positioning of the stimulus within the receptive field.

In sum, we conclude that humans show a clear advantage for detecting symmetry about the vertical axis but we do not see any clear neural correlate for this difference at the level of monkey IT cortex.

**SECTION 4. EFFECT OF SALIENCE ON SYMMETRY PERCEPTION**

In the main text, we have shown that symmetry perception can be predicted using distinctiveness as measured using visual search or using IT neurons. Specifically we have shown that symmetric objects that stand out more because of part summation elicit faster responses. Conversely asymmetric objects that stand out more because of part summation elicit slower responses. However there is a potential confound: the faster responses for distinctive symmetric objects could be explained by arguing that distinctive objects are salient and therefore elicit faster responses. However if this were true, distinctive asymmetric objects should also have elicited faster responses but they don’t. Thus, our pattern of results cannot be explained using simple bottom-up salience.

Here we performed an additional experiment to establish the effect of salience on symmetry perception. To this end, we repeated Experiment 3 of the main text on two sets of objects: high-contrast objects that were identical to those used in Experiment 3, and low-contrast (i.e. less salient) versions of the same objects. We predicted that making objects less salient would slow down responses to both symmetric and asymmetric objects. Note that this is qualitatively different from distinctiveness: objects that are more distinctive elicit slower responses if they are asymmetric and faster responses if they are symmetric. The critical difference between salience and distinctiveness is that while salience speeds up responses to both symmetric and asymmetric objects, distinctiveness speeds up responses specifically for symmetric but not asymmetric objects.

A total of 14 naïve human subjects (aged 22-40 years, seven female) participated in this experiment. The stimuli comprised 32 symmetric and 32 asymmetric objects used in experiment 3. We manipulated the saliency of objects by changing their brightness with respect to the background (background pixel intensity = 0). Objects in the high and low contrast sets had pixel intensities of 255 and 5 respectively. Thus, we created a set of 128 objects (64 high contrast + 64 low contrast) with equal number of symmetric and asymmetric objects. Subjects performed 4 correct trials for each object bringing the total number of correct trials to 512 (128 objects x 4 trials). High and low contrast objects were shown randomly interspersed within the experimental session.

Subjects were consistent in their performance as assessed using split-half correlation (r = 0.4, p = 0.01 for high-contrast images; and r = 0.32, p = 0.04 for low-contrast images). We assessed the effect of saliency on symmetry perception using an ANOVA on response times with subjects, symmetry and saliency (high- or low-contrast) as factors. We found significant main effects of all three factors (p < 0.00005). Subjects were slower on low-contrast objects compared to high-contrast objects (average response times: 423 ms and 465 ms for high- and low-contrast averaged across symmetric and asymmetric objects, p < 0.00005 for main effect of contrast in the same ANOVA; p = 0.61 for symmetry x contrast interaction). However, within each contrast group, symmetric objects were categorized faster than asymmetric objects (Figure S4; average response times: 407 ms and 439 ms for symmetric and asymmetric objects in high-contrast set; p < 0.005 for main effect of symmetry in an ANOVA with subject and symmetry as factors; 453 ms & 478 ms for symmetric and asymmetric low-contrast objects; p < 0.05 for main effect of symmetry in an ANOVA with subject and symmetry as factors). Additionally, response times were correlated between high- and low-contrast objects (r = 0.41, p = 0.02 for symmetric objects; and r = 0.47, p = 0.008 for asymmetric objects). Since we had collected visual search data for the same set of 32 symmetric and 32 asymmetric horizontal objects in Experiment 3 (main text), we asked how object distinctiveness explained the response times for high-contrast objects in this experiment. As expected, we found that distinctiveness speeded up responses to symmetric objects and slowed down responses to asymmetric objects (r = -0.36, p = 0.049 for symmetric objects; and r = 0.31, p = 0.08 for asymmetric objects).

To summarize, responses in symmetry detection become uniformly faster with increasing salience, whereas they become faster for symmetric objects and slower for asymmetric objects with increasing distinctiveness. Thus the effect of salience on symmetry perception is qualitatively different from the effect of distinctiveness.

**Figure S4 – Effect of salience on symmetry perception**

1. Bar plot showing the average response time in a symmetry judgement task for high- and low-contrast objects. Red and black bars correspond to symmetric and asymmetric objects respectively. Error bars indicate s.e.m calculated on objects.
